# Psychological outcomes and health-related quality of life changes in Chinese patients with moyamoya disease after revascularization

**DOI:** 10.3389/fsurg.2025.1573992

**Published:** 2025-04-11

**Authors:** Haijuan Liang, Ping Yuan, Tong Xu, Chao Jin, Cuiling Ji

**Affiliations:** Neurosurgical Intensive Care Unit, Nanjing Drum Tower Hospital Affiliated to Nanjing University Medical School, Nanjing City, Jiangsu, China

**Keywords:** editing moyamoya disease, revascularization, psychological outcomes, health-related quality of life, executive function

## Abstract

**Objectives:**

This study aimed to evaluate the psychological outcomes and changes in health-related quality of life (HRQOL) in Chinese patients with Moyamoya disease (MMD) following revascularization procedures.

**Methods:**

A total of 68 patients diagnosed with MMD and who underwent revascularization at Nanjing Drum Tower Hospital between January 2023 and January 2024 were retrospectively analyzed. Neuropsychological assessments, including the Trail Making Test, Chapuis Maze, Digit D2, Symptom Checklist-90 (SCL-90), Beck Depression Inventory-II (BDI-II), and the 36-item Short Form Health Survey (SF-36), were administered preoperatively and postoperatively at 3 months and 1 year. Statistical analysis was performed using SPSS version 29.0, with appropriate parametric or non-parametric tests applied based on data distribution.

**Results:**

Baseline characteristics revealed no significant differences between the Unremarkable and Impaired groups, confirming comparability. Postoperative improvements were observed in HRQOL across multiple domains, particularly in patients with preoperative impairments. Significant improvements were seen in physical functioning, general health, physical pain, emotional role function, and vitality (*P* < 0.05). Psychological outcomes also showed significant improvements, with reductions in aggressiveness, anxiety, and somatization (*P* < 0.001). Depression scores significantly decreased in 29.4% of patients (*P* < 0.001), and executive function, as measured by TMTA, TMTB, and Digit D2, also showed significant improvements in the impaired group (*P* < 0.001). However, patients without preoperative impairments exhibited no significant changes in any of the assessed domains.

**Conclusion:**

Revascularization significantly improves both psychological outcomes and HRQOL in Chinese patients with MMD, particularly in those with preoperative impairments. These findings highlight the importance of surgical intervention in enhancing both cognitive and psychological functioning in this patient population. Further prospective studies are warranted to confirm these results and explore long-term benefits.

## 1 Introduction

Moyamoya disease (MMD) is a chronic cerebrovascular disorder marked by progressive stenosis of the internal carotid arteries, leading to the formation of fragile collateral vessels in response to impaired blood flow (1, 2). Predominantly affecting East Asian populations, including Chinese individuals, MMD is associated with an increased risk of transient ischemic attacks (TIA), ischemic strokes, and intracerebral hemorrhage (3, 4). In addition to these serious physical consequences, MMD often results in significant cognitive impairment, emotional distress, and diminished health-related quality of life (HRQOL) (5, 6).

Revascularization surgery, specifically aimed at restoring cerebral perfusion and mitigating the risk of ischemic events, has been widely endorsed as the primary therapeutic approach for MMD (7). Studies have consistently demonstrated its efficacy in significantly reducing stroke recurrence (7, 8). However, recent research has increasingly focused on the broader neuropsychological and HRQOL outcomes following revascularization (9, 10). Cognitive impairments in MMD patients, particularly in domains such as executive function, attention, and memory, are prevalent due to chronic cerebral hypoperfusion (11). These deficits often exacerbate psychological issues, including depression, anxiety, and emotional instability, which are commonly reported in both pediatric and adult populations (6). While the surgical intervention successfully improves cerebral blood flow and reduces the risk of recurrent ischemic events, its effects on neurocognitive recovery are variable. Research indicates that cognitive function can improve post-surgery, especially in younger patients or those who undergo early intervention (9). However, long-term cognitive outcomes remain mixed, with some patients continuing to experience deficits in higher-order cognitive processes (10). Additionally, neuroimaging studies have shown that even after revascularization, regions of the brain previously affected by ischemia may not fully recover, leading to persistent cognitive challenges (12).

HRQOL in MMD patients is also notably impacted by both physical symptoms, such as fatigue and recurrent strokes, and cognitive and emotional challenges (13, 14). Post-revascularization HRQOL assessments show improvements in physical function, but mental health outcomes remain inconsistent (15). This underscores the importance of a multidisciplinary treatment approach for MMD, integrating cognitive rehabilitation and psychological interventions alongside surgery. However, the research specifically evaluating psychological outcomes and HRQOL changes following revascularization remains sparse, particularly in Chinese MMD patients. While many studies have focused on surgical and neurological outcomes, the effects of revascularization on cognitive function, emotional well-being, and overall quality of life in this demographic are underexplored. Considering the high prevalence of MMD in East Asian populations and the unique cultural and social factors influencing psychological health (3, 14, 16), it is essential to investigate these outcomes in Chinese patients.

This study aims to address this significant gap in the literature by systematically evaluating the psychological outcomes and HRQOL changes in Chinese MMD patients following revascularization surgery. Through detailed pre- and post-operative neuropsychological assessments, the study seeks to provide insights into the cognitive and emotional recovery associated with surgical intervention. By focusing on this understudied population, the research highlights the broader therapeutic potential of revascularization, offering important implications for improving overall patient management and quality of life in Chinese MMD patients.

## 2 Methods

### 2.1 Participants enrollment

This study retrospectively identified a group of Chinese patients diagnosed with MMD who subsequently underwent revascularization procedures. Patients were consecutively enrolled from the Nanjing Drum Tower Hospital Affiliated to Nanjing University Medical School between January 2023 and January 2024. A total of 68 participants met the eligibility criteria and were included in the final analysis. Selection criteria were based on established clinical and imaging guidelines. Specifically: (1) Clinical criteria: Patients exhibited ischemic symptoms [e.g., transient ischemic attack (TIA) or stroke] or progressive cognitive decline associated with MMD. (2) Imaging criteria: Diagnosis was confirmed via cerebral angiography, demonstrating progressive stenosis or occlusion of the internal carotid artery with characteristic collateral vessel formation. Additional evaluations, including functional MRI and H₂¹⁵O-PET with acetazolamide challenge, were used to assess cerebral perfusion status and determine surgical candidacy. Patients were excluded if they had secondary Moyamoya syndrome, severe comorbid conditions limiting surgery, or incomplete follow-up data. These criteria ensured a well-defined study population while maintaining clinical relevance. The study was approved by the Ethics Committee of the Nanjing Drum Tower Hospital Affiliated to Nanjing University Medical School. This study received ethics committee approval, ensuring compliance with ethical guidelines. Due to its retrospective design, obtaining individual informed consent was not feasible, and a waiver was granted per institutional and national regulations. To protect privacy, all data were anonymized before analysis, adhering to the Declaration of Helsinki. While informed consent was waived, patient rights remained safeguarded. In future studies, we recognize the value of prospective designs to obtain explicit consent and enhance ethical compliance. To ensure sufficient statistical power, we conducted a *post-hoc* power analysis. Based on observed effect sizes (Cohen's d ranging from 0.57 to 4.59), the required sample sizes to achieve 80% power varied between 3 and 26 participants, except for Chapuis Maze, which exhibited a negligible effect size. Given that our study included 68 participants, the power is more than sufficient for detecting significant changes in key outcomes. However, for detecting very small effects, particularly in spatial memory (Chapuis Maze), a much larger sample may be required in future research.

### 2.2 Neuropsychological assessments

Certified neuropsychologists conducted assessments covering executive function, psychological well-being, depression, and health-related quality of life (HRQOL). The NPAs included the Trail Making Test A (TMTA) and B (TMTB) for executive function, Chapuis Maze (CM) for spatial navigation, and Digit D2 (D2) for attention and processing speed. Psychological outcomes were measured with the Symptom Checklist-90 (SCL-90), depression with the Beck Depression Inventory-II (BDI-II), and HRQOL with the 36-item Short Form Health Survey (SF-36). The cutoff values of function impairment are defined based on previous report (17, 18). Preoperative imaging used cerebral panangiography, functional MRI, and H2 15 O-PET with acetazolamide challenge to assess disease severity and cerebral perfusion. Multiterritorial revascularization was performed in stages as needed. Postoperative assessments, including MRI, occurred 3 months after surgery, and follow-up assessments were conducted at 1 year to assess cognitive and psychological changes.

### 2.3 Statistical analysis

The data analysis and statistical evaluations were conducted using the latest version of SPSS software (SPSS 29.0). To determine whether the parametric data conformed to a normal distribution, the Shapiro–Wilk test was applied. For data that adhered to parametric assumptions, a paired t-test was performed with a 95% confidence interval. For non-parametric data, the Wilcoxon signed-rank test was employed. The significance level was set at 0.05 for all statistical analyses.

## 3 Results

### 3.1 Baseline characteristics

Baseline characteristics showed no significant differences between the Unremarkable and Impaired groups, confirming comparability (Table 1). The mean age was 38.96 ± 9.26 years in the Unremarkable group and 41.25 ± 8.77 years in the Impaired group (*P* = 0.349), with similar BMI (24.43 ± 2.86 vs. 24.49 ± 3.25, *P* = 0.941) and IQ (102.15 ± 9.47 vs. 101.10 ± 7.73, *P* = 0.664). Sex distribution was female-dominant in both groups (72.9% vs. 55.0%, *P* = 0.248). Current smoking was lower in the Impaired group (15.0%) than in the Unremarkable group (31.2%), while non-smoking rates were higher (65.0% vs. 47.9%) (*P* = 2.191). Hypertension (*P* = 0.402) and diabetes (*P* = 0.354) were evenly distributed. Education levels were comparable, with 43.8% vs. 40.0% having a high school education (*P* = 0.339). Alcohol consumption patterns were similar (*P* = 0.452), with occasional drinking more common in the Impaired group (55.0% vs. 39.6%). Overall, the lack of significant differences (*P* > 0.05) confirms well-matched groups, ensuring a valid comparison of postoperative outcomes.

**Table 1:** Baseline characteristics of unremarkable and impaired groups.

Clinical indices	Unremarkable	Impaired	*P*
Age	38.96 ± 9.26	41.25 ± 8.77	0.349
BMI	24.43 ± 2.86	24.49 ± 3.25	0.941
IQ	102.15 ± 9.47	101.10 ± 7.73	0.664
Sex			0.248
Male	13 (27.1)	9 (45.0)	
Female	35 (72.9)	11 (55.0)	
Smoking Status			2.191
Current	15 (31.2)	3 (15.0)	
Former	10 (20.8)	4 (20.0)	
Never	23 (47.9)	13 (65.0)	
Hypertension			0.402
Yes	36 (75.0)	13 (65.0)	
No	12 (25.0)	7 (35.0)	
Diabetes			0.354
Yes	42 (87.5)	19 (95.0)	
No	6 (12.5)	1 (5.0)	
Education Level			0.339
High school	21 (43.8)	8 (40.0)	
University	27 (56.2)	12 (60.0)	
Alcohol Consumption			0.452
None	20 (41.7)	7 (35.0)	
Occasional	19 (39.6)	11 (55.0)	
Regular	9 (18.8)	2 (10.0)	

Preoperative characteristics revealed that 67.6% of the cohort were female, with an average age of 41.3 years (SD = 15.1). BMI categorized 26.5% of patients as overweight, 14.7% as obese, and 58.8% with normal weight. Smoking was active in 29.4%, with 22.1% as former smokers, and 48.5% as non-smokers. Alcohol consumption was regular in 14.7%, occasional in 36.8%, and absent in 48.5%. Physical inactivity was noted in 51.5%, and 52.9% had university degrees. Employment status showed 73.5% employed. Initial symptoms included TIA (27.9%), stroke (36.8%), ICH (11.8%), and minor symptoms (20.6%). Imaging revealed bilateral moyamoya in 83.8%. A total of 114 MCA bypasses and 28 ACA bypasses were performed with high patency. Postoperative outcomes were favorable, with 88.2% having no complications, and limited recurrence of TIA (7.4%) and stroke (2.9%). Detailed information was shown in Table 2.

**Table 2:** Baseline characteristics of general patients.

Characteristic	Total (*n* = 68)
Sex	
Female	46 (67.6%)
Male	22 (32.4%)
Age (years)	41.3 ± 15.1
BMI	24.5 ± 3.2
Overweight	18 (26.5%)
Obesity	10 (14.7%)
Normal	40 (58.8%)
Smoking History	
Current	20 (29.4%)
Former	15 (22.1%)
Never	33 (48.5%)
Alcohol History	
Regular	10 (14.7%)
Occasional	25 (36.8%)
None	33 (48.5%)
Exercise Habits	
Sedentary	35 (51.5%)
Active	33 (48.5%)
Educational Background	
High School	32 (47.1%)
University	36 (52.9%)
Employment Status	
Employed	50 (73.5%)
Unemployed	18 (26.5%)
Family History of Moyamoya	8 (11.8%)
Handedness (EHI)	
Right	53 (77.9%)
Left	5 (7.4%)
Ambidexter	10 (14.7%)
Initial Symptoms/Manifestation	
TIA	19 (27.9%)
Stroke	25 (36.8%)
ICH	8 (11.8%)
Minor (headache, performance deficit)	14 (20.6%)
Incidental	2 (2.9%)
Moyamoya Disease Type	
Unilateral left	4 (5.9%)
Unilateral right	7 (10.3%)
Bilateral	57 (83.8%)
Infarction Location (MRI)	
ACA left	30 (44.1%)
ACA right	32 (47.1%)
MCA left	51 (75.0%)
MCA right	54 (79.4%)
PCA left	0 (0%)
PCA right	0 (0%)
IQ (MWT-B)	101.7 ± 13.1
Impaired (<85)	5 (7.4%)
Normal (≥85)	63 (92.6%)
Comorbid Conditions	
Diabetes	8 (11.8%)
Hypertension	18 (26.5%)
Revascularization	
MCA Bypass	114 (total)
Direct left/right	33/33
Combined direct + indirect left/right	22/26
ACA Bypass	28 (total)
indirect	13/15
Bypass Patency	
MCA	111 (97.4%)
ACA	28 (100%)
Intraoperative Complications	
None	62 (91.2%)
Minor	5 (7.4%)
Major	1 (1.5%)
Post-Operative Complications	
None	60 (88.2%)
Minor	6 (8.8%)
Major	2 (2.9%)
Medication Use Post-Op	
Anticoagulants	32 (47.1%)
Statins	28 (41.2%)
Recurrence of Symptoms Post-Op	
TIA	5 (7.4%)
Stroke	2 (2.9%)
None	61 (89.7%)

BMI, body mass index; EHI, edinburgh handedness inventory; TIA, transient ischemic attack; ICH, intracerebral hemorrhage; MRI, magnetic resonance imaging; ACA, anterior cerebral artery; MCA, middle cerebral artery; PCA, posterior cerebral artery; IQ, intelligence quotient; MWT-B, multiple choice word test (version B).

### 3.2 Postoperative improvements in health-related quality of life evaluated by SF-36

Following revascularization, significant improvements in health-related quality of life (HRQOL) were observed across multiple domains in Chinese patients with moyamoya disease, as measured by the SF-36 scale. Among those classified as impaired preoperatively, physical functioning improved in 27.9% of patients, with scores rising from −2.59 ± 0.45 to −1.06 ± 0.50 (*P* < 0.05). General health also showed a notable increase, with scores improving from −2.89 ± 0.83 to −1.02 ± 1.04 in 47.1% of impaired patients (*P* < 0.05). Physical pain and physical role function improved significantly in 22.1% and 41.2% of patients with impairments, respectively, with physical pain scores improving from −1.94 ± 0.30 to −0.50 ± 0.43 (*P* < 0.05), and physical role function showing substantial gains from −2.29 ± 0.43 to −0.84 ± 0.50 (*P* < 0.05). Psychological well-being and emotional role function similarly showed improvements, with psychological well-being scores rising in 33.8% of impaired patients from −2.06 ± 0.35 to −0.59 ± 0.46 (*P* < 0.05), and emotional role function improving in 36.8% of patients from −3.18 ± 0.46 to −1.50 ± 0.64 (*P* < 0.05). Social functioning improved in 27.9% of those classified as impaired, with scores rising from −2.58 ± 0.24 to −1.12 ± 0.38 (*P* < 0.05), while vitality saw the greatest enhancement, with scores increasing from −1.33 ± 0.21 to −0.04 ± 0.41 in 38.2% of impaired patients (*P* < 0.05). In contrast, patients classified as unremarkable preoperatively did not exhibit significant changes postoperatively. Detailed information is presented in Table 3.

**Table 3:** Postoperative improvements in health-related quality of life (HRQOL) in Chinese patients with moyamoya disease, evaluated by the SF-36 scale.

Function state	Physical functioning				Effect size	General health				Effect size
	*n* (%)	Preoperative	Postoperative	*P* value		*n* (%)	Preoperative	Postoperative	*P* value	
Impaired	19 (27.9)	−2.59 ± 0.45	−1.06 ± 0.50	<0.001	−3.22	32 (47.1)	−2.89 ± 0.83	−1.02 ± 1.04	<0.001	−1.99
Unremarkable	49 (72.1)	−0.1 ± 0.58	−0.09 ± 0.60	0.545	−0.02	36 (52.9)	0.22 ± 0.44	0.23 ± 0.44	0.455	−0.02
Function state	Physical Pain					Physical Role Function				
	*n* (%)	Preoperative	Postoperative	*P* value	Effect size	*n* (%)	Preoperative	Postoperative	*P* value	Effect size
Impaired	15 (22.1)	−1.94 ± 0.30	−0.50 ± 0.43	<0.001	−3.88	28 (41.2)	−2.29 ± 0.43	−0.84 ± 0.50	<0.001	−3.11
Unremarkable	53 (77.9)	0.47 ± 0.30	0.50 ± 0.37	0.121	−0.09	40 (58.8)	−0.32 ± 0.42	−0.17 ± 0.57	0.053	−0.3
Function state	Psychological Well-being					Emotional Role Function				
	*n* (%)	Preoperative	Postoperative	*P* value	Effect size	*n* (%)	Preoperative	Postoperative	*P* value	Effect size
Impaired	23 (33.8)	−2.06 ± 0.35	−0.59 ± 0.46	<0.001	−3.6	25 (36.8)	−3.18 ± 0.46	−1.50 ± 0.64	<0.001	−3.01
Unremarkable	45 (66.2)	0.17 ± 0.25	0.22 ± 0.29	0.074	−0.18	43 (63.2)	0.21 ± 0.11	0.22 ± 0.13	0.092	−0.08
Function state		Social Functioning					Vitality			
	*n* (%)	Preoperative	Postoperative	*P* value	Effect size	*n* (%)	Preoperative	Postoperative	*P* value	Effect size
Impaired	19 (27.9)	−2.58 ± 0.24	−1.12 ± 0.38	<0.001	−4.59	26 (38.2)	−1.33 ± 0.21	−0.04 ± 0.41	<0.001	−3.96
Unremarkable	49 (72.1)	0.27 ± 0.20	0.14 ± 0.48	0.063	0.35	42 (61.8)	−0.30 ± 0.37	−0.18 ± 0.55	0.056	−0.26

This table presents preoperative and postoperative scores for physical and mental health-related quality of life (HRQOL) domains, assessed using the SF-36 scale. The Impaired group exhibited significant improvements across all domains (*P* < 0.001), with large effect sizes, whereas the Unremarkable group showed minimal or non-significant changes. Scores are presented as mean ± standard deviation, with *P*-values reflecting paired *t*-test comparisons between preoperative and postoperative assessments. Effect sizes indicate the magnitude of change, with negative values denoting improvement in HRQOL metrics.

### 3.3 Postoperative improvements in health-related quality of life evaluated by SCL-90

Meanwhile, significant improvements in psychological outcomes were observed after revascularization, as measured by the SCL-90 scale. Among patients classified as impaired preoperatively, aggressiveness improved in 19.1%, with scores decreasing from 66.26 ± 2.91 to 64.21 ± 2.85 (*P* < 0.001). Anxiety also showed a notable decrease in 29.4% of impaired patients, with scores improving from 67.23 ± 4.33 to 64.30 ± 4.63 (*P* < 0.001). Obsessiveness and paranoid thinking demonstrated similar improvements. Obsessed patients (39.7%) showed a decrease in scores from 65.91 ± 1.45 to 62.00 ± 2.97 (*P* < 0.001), while those with paranoid thinking (8.8%) improved from 62.02 ± 0.95 to 58.50 ± 4.25 (*P* = 0.05). Insecurity improved in 7.4% of patients, with scores dropping from 67.94 ± 1.82 to 65.47 ± 3.55, which is not statistically significant (*P* = 0.05). Somatization showed a marked decrease in 32.4% of impaired patients, with scores improving from 64.16 ± 2.62 to 56.61 ± 3.56 (*P* < 0.001). Psychoticism improved significantly in 41.2% of patients, with scores decreasing from 63.33 ± 1.72 to 56.09 ± 2.09 (*P* < 0.001). Phobic-anxiety showed a slight improvement in 22.1% of patients, with scores decreasing from 65.91 ± 2.60 to 64.18 ± 3.46, though this change was not statistically significant (*P* = 0.051). In contrast, patients classified as unremarkable preoperatively showed no significant changes postoperatively. Detailed information was presented in Table 4.

**Table 4:** Postoperative improvements in psychological outcomes in Chinese patients with moyamoya disease, evaluated by the SCL-90 scale.

Function state	Aggressiveness				Effect size	Anxiety				Effect size
	*n* (%)	Preoperative	Postoperative	*P* value		*n* (%)	Preoperative	Postoperative	*P* value	
Impaired	13 (19.1)	66.26 ± 2.91	64.21 ± 2.85	<0.001	0.71	20 (29.4)	67.23 ± 4.33	64.30 ± 4.63	<0.001	0.65
Unremarkable	55 (80.9)	49.81 ± 3.00	50.05 ± 2.96	0.053	−0.08	48 (70.6)	52.72 ± 2.91	52.06 ± 3.88	0.052	0.19
Function state	Obsessiveness					Paranoid Thinking				
	*n* (%)	Preoperative	Postoperative	*P* value	Effect size	*n* (%)	Preoperative	Postoperative	*P* value	Effect size
Impaired	27 (39.7)	65.91 ± 1.45	62.00 ± 2.97	<0.001	1.67	6 (8.8)	62.02 ± 0.95	58.50 ± 4.25	0.05	1.14
Unremarkable	41 (60.3)	47.11 ± 4.29	46.86 ± 4.25	0.057	0.06	62 (91.2)	45.16 ± 2.89	44.89 ± 2.98	0.051	0.09
Function state	Insecurity					Somatization				
	*n* (%)	Preoperative	Postoperative	*P* value	Effect size	*n* (%)	Preoperative	Postoperative	*P* value	Effect size
Impaired	5 (7.4)	67.94 ± 1.82	65.47 ± 3.55	0.05	0.88	22 (32.4)	64.16 ± 2.62	56.61 ± 3.56	<0.001	2.42
Unremarkable	63 (92.6)	46.20 ± 3.85	46.27 ± 3.84	0.589	−0.02	46 (67.6)	47.03 ± 2.97	46.87 ± 2.93	0.051	0.05
Function state	Psychoticism					Phobic-Anxiety				
	*n* (%)	Preoperative	Postoperative	*P* value	Effect size	*n* (%)	Preoperative	Postoperative	*P* value	Effect size
Impaired	28 (41.2)	63.33 ± 1.72	56.09 ± 2.09	<0.001	3.78	15 (22.1)	65.91 ± 2.60	64.18 ± 3.46	0.051	0.57
Unremarkable	40 (58.8)	48.32 ± 4.41	48.16 ± 4.48	0.387	0.04	53 (77.9)	49.93 ± 3.64	49.68 ± 3.75	0.115	0.07

This table summarizes the changes in psychological distress symptoms, measured using the Symptom Checklist-90 (SCL-90) scale. The Impaired group demonstrated significant reductions in aggressiveness, anxiety, obsessiveness, and somatization symptoms (*P* < 0.05), while the Unremarkable group exhibited only marginal changes. Scores are expressed as mean ± standard deviation, with *P*-values from paired t-tests assessing preoperative and postoperative differences. Effect sizes quantify the magnitude of psychological symptom improvement, with higher positive values indicating greater reductions in distress.

### 3.4 Postoperative improvements in depression

After revascularization, participants experienced marked reductions in depressive symptoms, as assessed by the BDI-II and the depression component of the SCL-90 scale. Among the 29.4% of patients (*n* = 20) classified as impaired preoperatively, BDI-II scores significantly decreased from 19.45 ± 3.03 to 12.01 ± 3.59 postoperatively (*P* < 0.001). In contrast, no significant change was observed in the 70.6% of patients (*n* = 48) classified as unremarkable (*P* = 0.051). Similarly, the depression item of the SCL-90 improved in 32.4% of impaired patients (*n* = 22), with scores decreasing from 64.60 ± 2.67 to 59.86 ± 3.27 postoperatively (*P* < 0.001). No significant changes were noted in the 67.6% of patients (*n* = 46) with unremarkable preoperative scores (*P* = 0.494). These results indicate that revascularization significantly reduces depressive symptoms in patients with preoperative impairments. Detailed results are presented in Table 5.

**Table 5:** Postoperative improvements in depressive symptoms in Chinese patients with moyamoya disease, evaluated by the BDI-II and SCL-90 scales.

Function state	BDI-II				Effect size	SCL-90				Effect size
	*n* (%)	Preoperative	Postoperative	*P* value		*n* (%)	Preoperative	Postoperative	*P* value	
Impaired	20 (29.4)	19.45 ± 3.03	12.01 ± 3.59	<0.001	2.24	22 (32.4)	64.60 ± 2.67	59.86 ± 3.27	<0.001	1.59
Unremarkable	48 (70.6)	5.25 ± 1.46	5.07 ± 1.61	0.051	0.12	46 (67.6)	13.69 ± 3.45	13.81 ± 3.90	0.494	−0.03

This table reports the impact of revascularization on depressive symptoms, using Beck Depression Inventory-II (BDI-II) and the SCL-90 depression subscale. The Impaired group exhibited substantial improvement in depressive symptoms (*P* < 0.001), with large effect sizes (Cohen's d > 1.5). The Unremarkable group showed no statistically significant changes (*P* > 0.05). Data are presented as mean ± standard deviation, with *P*-values derived from paired t-tests. Effect sizes reflect the degree of improvement, with positive values denoting reductions in depressive symptom severity.

### 3.5 Postoperative improvements in executive function

In this study, executive function was evaluated in Chinese patients with moyamoya disease before and after revascularization using the Trail Making Test A (TMTA), Trail Making Test B (TMTB), Chapuis Maze (CM) score, and Digit D2 (D2) score. Among patients classified as impaired preoperatively, TMTA scores improved significantly in 45.6% of patients, with values rising from −3.08 ± 1.11 to −1.10 ± 1.21 postoperatively (*P* < 0.001). Similarly, TMTB scores improved in 47.1% of impaired patients, increasing from −2.31 ± 0.67 to −0.38 ± 0.72 (*P* < 0.001). Chapuis Maze performance showed a modest, non-significant improvement in 44.1% of impaired patients, with scores increasing from 6.19 ± 3.10 to 6.54 ± 3.19 (*P* = 0.094). In contrast, significant gains were observed in the Digit D2 scores, with 42.6% of impaired patients improving from 81.44 ± 2.06 to 86.54 ± 3.06 postoperatively (*P* < 0.001). Patients classified as unremarkable preoperatively did not experience notable changes postoperatively, as reflected by non-significant differences in scores for TMTA (*P* = 0.597), TMTB (*P* = 0.7), CM (*P* = 0.641), and D2 (*P* = 0.068). Detailed information is presented in Table 6.

**Table 6:** Postoperative improvements in executive function in Chinese patients with moyamoya disease, evaluated by TMTA, TMTB, chapuis maze, and digit D2.

Function state	TMTA score				Effect size	TMTB score				Effect size
	*n* (%)	Preoperative	Postoperative	*P* value		*n* (%)	Preoperative	Postoperative	*P* value	
Impaired	31 (45.6)	−3.08 ± 1.11	−1.10 ± 1.21	<0.001	−1.71	32 (47.1)	−2.31 ± 0.67	−0.38 ± 0.72	<0.001	−2.78
Unremarkable	37 (54.4)	0.19 ± 0.45	0.16 ± 0.52	0.597	0.06	36 (52.9)	−0.07 ± 0.58	−0.04 ± 0.81	0.7	−0.04
Function state	Chapuis Maze (CM) Score					Digit D2 (D2) Score				
	*n* (%)	Pre	Post	*P* value	Effect size	*n* (%)	Pre	Post	*P* value	Effect size
Impaired	30 (44.1)	6.19 ± 3.10	6.54 ± 3.19	0.094	−0.11	29 (42.6)	81.44 ± 2.06	86.54 ± 3.06	<0.001	−1.96
Unremarkable	38 (55.9)	69.15 ± 12.81	69.39 ± 13.48	0.641	−0.02	39 (57.4)	99.46 ± 2.91	99.96 ± 2.99	0.068	−0.17

## 4 Discussion

Moyamoya disease (MMD) is a chronic cerebrovascular disorder that predominantly affects East Asian populations, including Chinese individuals, leading to significant risks of stroke, cognitive impairment, and diminished HRQOL (19, 20). While revascularization surgery has been established as an effective intervention to restore cerebral perfusion and reduce ischemic events, its impact on neuropsychological recovery and HRQOL remains variable. This study aimed to evaluate the psychological outcomes and HRQOL in Chinese patients with MMD following revascularization surgery. The findings demonstrate significant improvements across multiple domains, particularly in those patients who exhibited preoperative impairments in executive function, psychological well-being, and HRQOL.

The results indicated substantial improvements in physical functioning, general health, and emotional role functioning among patients with preoperative impairments. Specifically, 27.9% of patients exhibited enhanced physical functioning, and 47.1% showed improved general health, consistent with findings from previous studies that have linked improved cerebral perfusion with better physical outcomes post-revascularization (7, 21). The improvements in physical pain and role function also align with research suggesting that revascularization alleviates the physical burden of recurrent ischemic events (22, 23). However, unlike studies reporting mixed results in mental health domains, this study found significant gains in psychological well-being, emotional role functioning, and vitality. This discrepancy may be attributed to differences in cultural perceptions of health and the specific neuropsychological interventions provided in the Chinese healthcare context, which emphasize both physical recovery and emotional support (24, 25). Interestingly, patients classified as “unremarkable” preoperatively did not show significant changes postoperatively, reinforcing the hypothesis that surgical interventions primarily benefit those with pre-existing deficits. This outcome is consistent with the literature suggesting that cognitive recovery is most pronounced in patients who exhibit measurable impairments prior to revascularization (10, 26).

Moreover, the improvements in psychological outcomes, particularly reductions in anxiety, obsessiveness, aggressiveness, and somatization, suggest that revascularization has a profound impact on emotional regulation in MMD patients. This may be partly explained by the restoration of cerebral blood flow to areas of the brain responsible for emotional control, such as the prefrontal cortex and amygdala (27, 28). The observed decrease in depression symptoms, as reflected in both the BDI-II and SCL-90, is noteworthy. Depression is a common comorbidity in MMD, often exacerbated by chronic hypoperfusion (29, 30). The reduction in depressive symptoms in 29.4% of patients is consistent with research highlighting the psychological benefits of revascularization in cerebrovascular disorders (31). These findings support the notion that improving cerebral perfusion can have a cascading effect on psychological health, reducing the emotional distress that often accompanies chronic illness. Nevertheless, the limited improvements in phobic-anxiety and insecurity scores indicate that not all psychological symptoms are equally responsive to surgical intervention. This suggests the need for adjunctive therapies, such as cognitive-behavioral therapy (CBT), to address persistent anxiety and insecurity (32, 33).

The significant postoperative improvements in executive function, as measured by the Trail Making Test (TMT) and Digit D2 tests, underscore the cognitive benefits of revascularization in MMD patients. Executive dysfunction in MMD is thought to result from chronic cerebral hypoperfusion, particularly in frontal lobe regions (23, 34). The observed improvements in TMTA and TMTB scores in 45.6% and 47.1% of impaired patients, respectively, suggest that revascularization can restore higher-order cognitive processes such as attention, task-switching, and problem-solving. These findings are consistent with studies that have shown similar improvements in cognitive function following revascularization, particularly in younger patients or those undergoing early intervention (15, 35–37). However, the lack of significant change in the Chapuis Maze (CM) test highlights a potential limitation in the extent of cognitive recovery. Spatial navigation deficits, which are often linked to hippocampal damage, may not fully resolve despite improved blood flow, as the hippocampus is highly susceptible to ischemic injury (38–40). This underscores the importance of neuroprotective strategies to preserve cognitive function during the chronic phases of MMD.

This study is the first to systematically investigate the psychological outcomes and HRQOL changes in Chinese patients with MMD following revascularization surgery, addressing a significant gap in the current literature. By applying stringent inclusion and exclusion criteria, this study excluded patients with significant psychiatric comorbidities and advanced cognitive impairments, ensuring the homogeneity of the study sample and enhancing the validity and reliability of the findings. Additionally, the use of multiple validated psychological and cognitive assessment tools, such as the SCL-90, BDI-II, SF-36, and Trail Making Test (TMT), allowed for a comprehensive evaluation of changes in patients' psychological and cognitive functions, ensuring a more accurate understanding of the impact of surgery on their quality of life and mental health.

## 5 Limitations

However, this study has several limitations. This retrospective study relies on past records, introducing selection bias as only patients who underwent surgery were included, likely those with severe symptoms or better adherence. This limits generalizability, especially to milder cases or non-surgical patients. To mitigate bias, we defined clear inclusion/exclusion criteria and controlled for confounders where possible. However, prospective cohorts or RCTs are needed for stronger causal inference. As a single-center study, institutional/regional factors may further impact external validity. Future multi-center studies could improve generalizability. Additionally, the one-year follow-up may not capture long-term cognitive and psychological outcomes, necessitating further studies to assess whether improvements are sustained or if cognitive decline recurs.

This study employs the SCL-90 and BDI-II to assess psychological distress in Chinese Moyamoya patients, but cultural differences in emotional expression may affect their accuracy. While Western populations primarily report emotional symptoms like sadness, Chinese individuals often express distress through somatic complaints (e.g., fatigue, dizziness), potentially leading to underreporting of depression. Though validated in Chinese populations, these tools may not fully capture culturally specific distress patterns—for instance, lower cognitive-affective scores but higher somatic symptom reports. To improve cultural sensitivity, future studies should consider China-specific scales (e.g., SDS), qualitative interviews, or structured clinical assessments. While SCL-90 and BDI-II remain valid, their cultural adaptability is a limitation, warranting further refinement for Chinese patients.

## 6 Conclusion

In summary, this study demonstrates that revascularization surgery leads to significant improvements in HRQOL, psychological well-being, and executive function in Chinese patients with MMD, particularly among those with preoperative impairments. These findings provide strong evidence for the broader therapeutic benefits of revascularization beyond stroke prevention, highlighting its potential to alleviate the cognitive and emotional burden of MMD. Further research is needed to explore the long-term sustainability of these benefits and the potential role of adjunctive therapies in optimizing patient outcomes.

## Data Availability

The raw data supporting the conclusions of this article will be made available by the authors, without undue reservation.
